# Using virtual reality to induce multi-trial inattentional blindness despite trial-by-trial measures of awareness

**DOI:** 10.3758/s13428-024-02401-8

**Published:** 2024-04-09

**Authors:** Rony Hirschhorn, Dan Biderman, Natalie Biderman, Itay Yaron, Rotem Bennet, Meir Plotnik, Liad Mudrik

**Affiliations:** 1 Sagol School of Neuroscience, Tel-Aviv University, Ramat Aviv, POB 39040, 6997801 Tel Aviv, Israel; 2 Mortimer B. Zuckerman Mind, Brain, Behavior Institute, Columbia University, New York, NY USA; 3 Department of Psychology, Columbia University, New York, NY USA; 4 School of Psychological Sciences, Tel Aviv University, Tel Aviv, Israel; 5 Center of Advanced Technologies in Rehabilitation, Sheba Medical Center, Ramat Gan, Israel; 6 Department of Physiology and Pharmacology, Faculty of Medicine, Tel Aviv University, Tel Aviv, Israel

**Keywords:** Inattentional blindness, Virtual reality, Unconscious processing, Ecological research

## Abstract

Unconscious processing has been widely examined using diverse and well-controlled methodologies. However, the extent to which these findings are relevant to real-life instances of information processing without awareness is limited. Here, we present a novel inattentional blindness (IB) paradigm in virtual reality (VR). In three experiments, we managed to repeatedly induce IB while participants foveally viewed salient stimuli for prolonged durations. The effectiveness of this paradigm demonstrates the close relationship between top-down attention and subjective experience. Thus, this method provides an ecologically valid setup to examine processing without awareness.

**Supplementary information:**

The online version contains supplementary material available at 10.3758/s13428-024-02401-8.

## Introduction

What is the function of consciousness? Different answers have been suggested to this question, from assigning different high-level functions to consciousness (e.g., working memory: Baars & Franklin, 2003; Baddeley, 2003; integration of information: Hirschhorn et al., 2021; Mudrik et al., 2014; chaining mental operations: Sackur & Dehaene, 2009; and flexibility: Searle, 1992) to portraying consciousness as devoid of any function whatsoever (e.g., Hassin, 2013; Huxley, 1874). Such claims typically rely on empirical findings, where specific processes are shown to take place without awareness, or to require awareness (for a recent review, see Mudrik & Deouell, 2022). To obtain such findings, researchers have developed a myriad of elegant psychophysical methods for manipulating consciousness (for reviews, see Breitmeyer, 2015; Kim & Blake, 2005).

These methods involve manipulating factors such as the intensity, duration, or timing of a stimulus presentation to render it invisible, and measuring the effect it nevertheless exerts on behavior, or on some physiological/neural responses. However, most of these paradigms produce perceptual states that are very distant from everyday conscious and unconscious processing. For example, methods that present a stimulus that is masked or obscured by other stimuli (masking: Marcel, 1983), or ones where a different stimulus is presented to each eye (binocular rivalry: Breese, 1909; continuous flash suppression: Tsuchiya & Koch, 2005), elicit states that are not representative of the ones people experience outside the laboratory.

This gap between the phenomenon of interest and its operationalization raises concerns with respect to the validity of past findings. In the abovementioned psychophysical manipulations, the operationalized concepts are different types of unconscious processing (e.g., can we integrate information without awareness? Hirschhorn et al., 2021; can we read without awareness? Sklar et al., 2012; can we process emotional stimuli without awareness? Smith, 2012). However, as the states induced by the psychophysical manipulations are unique to lab-based settings, the probed processes might be idiosyncratic to the specific manipulation (Dubois & Faivre, 2014; Peremen & Lamy, 2014). In that case, the operationalization might only partially capture the true nature of unconscious processes, probing situations that may be too distant from the actual processes they are supposed to mimic (diminishing the paradigm’s construct validity: Cronbach & Meehl, 1955). This further raises the concern that the results might not generalize to real-life settings and circumstances (affecting their ecological validity: Andrade, 2018, as well as their overall external validity: Bracht & Glass, 1968). This concern about the extent to which a study’s results are indeed relevant to real-life situations has been acknowledged as an important factor in psychology and neuroscience studies in general (Shamay-Tsoory & Mendelsohn, 2019; van Atteveldt et al., 2018), but might be especially relevant to studies of unconscious versus conscious processes, where the manipulations yield states that are very unique and uncommon, with hardly any attempt to get closer to the way unconscious processes unfold in day-to-day lives.

A notable exception is the manipulation of consciousness via attention, which seems more representative of realistic instances. In such attentional manipulations, like load-induced blindness (Macdonald & Lavie, 2008), change blindness (CB: Simons & Levin, 1997), and inattentional blindness (IB: Becklen & Cervone, 1983; Mack & Rock, 1998), participants fail to notice unexpected objects because their attention is engaged elsewhere. This top-down manipulation does not necessitate any physical degradation of the stimuli: salient, dynamic stimuli can be presented for prolonged durations, and still go completely unnoticed (Simons & Chabris, 1999).

However, using IB to manipulate consciousness has its downsides: First, some have suggested that the failure to report the critical stimulus might reflect a memory failure (“inattentional amnesia”: Wolfe, 1999) rather than a perceptual one (see also Lamme, 2006). Because participants are probed only after the stimulation has ended, it may be that despite having actually consciously perceived the stimulus, by the time they are asked about it, their fleeting conscious experience of it is forgotten. This criticism has been directly addressed though, by showing that manipulating the time between the stimulus and the probe does not affect the likelihood of noticing it (Becklen & Cervone, 1983). Even more convincing is the demonstration that IB can occur even when the report of the unexpected event is given while the event is still on the screen (Ward & Scholl, 2015). Thus, it seems less likely that the failure to report the critical stimulus in an IB paradigm is memory-driven (albeit still possible: Wang et al., 2021).

Still, the use of IB as a means to study unconscious processes has been relatively limited (Pitts et al., 2012; Thakral, 2011). The main reason is the apparent inability to effectively repeat IB over many trials, while measuring awareness on a trial-by-trial basis; for example, once participants are asked about the invisible gorilla (Simons & Chabris, 1999), they see it in subsequent presentations. Consequently, many IB studies either include only one critical trial (or two trials: Murphy & Greene, 2016; Potchen, 2006; Simons & Schlosser, 2017; Ward & Scholl, 2015) or present multiple trials, yet measuring awareness only at the end of the session. Relying on a single trial is problematic for the study of unconscious processing, because multiple presentations are needed to study the difference between seen and unseen stimuli (Hutchinson, 2019). Similarly, measuring awareness post hoc does not allow the exclusion of trials in which the stimulus might have been consciously experienced, potentially contaminating the results with conscious trials. Additionally, such a report might reflect a memory failure rather than a perceptual one (Wolfe, 1999), as discussed above.

Another issue is that, unlike the objective and subjective methods that are commonly used in psychophysical manipulations (Sandberg et al., 2010), the questions comprising the awareness assessment in IB studies vary: while some include an objective measure of awareness (e.g., Thakral, 2011), others do not (e.g., Murphy & Greene, 2016), and the subjective question also varies in its specificity (e.g., Pitts et al., 2012: “Did you notice any patterns…? If you did see any patterns, please describe (or draw) what you saw…”; Ward & Scholl, 2015: “anything...that was different from the first three trials?”). This creates difficulty in comparing the IB paradigm to other manipulations of awareness and differentiating conscious from unconscious processes. Hence, a multi-trial IB paradigm with a trial-by-trial assessment of awareness is needed to study conscious versus unconscious processing in a more ecological manner. Such a paradigm has not been introduced thus far, probably given the challenge of creating a task that would engage attention substantially enough to induce IB despite being repeatedly probed about the critical stimulus, on a trial-by-trial basis.

Here, we overcome this challenge using a head-mounted virtual reality (VR) environment, which provides an engaging, ecological and interactive setup (compared to experiments in which participants respond to computerized, two-dimensional stimuli; Wilson & Soranzo, 2015). The choice of VR for this task was motivated by two main reasons. First, VR environments are more immersive compared to computerized paradigms (Shamay-Tsoory & Mendelsohn, 2019): unlike contents presented on computer screens (which can appear either 2D or 3D), the worn headset provides a wide field of view, which maximizes immersion and sense of presence (Duh et al., 2001). An additional feature of the VR setup that increases immersion is the isolation from the ‘real’ lab environment. Also, with the use of headsets, this field of view adjusts based on observers’ head movements, mimicking everyday vision and engaging observers in a more realistic manner. Indeed, a recent systematic review comparing VR and computerized experiments to real-world environments found that VR shares more similarities with the real world compared to computer screen contents (Hepperle & Wölfel, 2023), further supporting the external validity of this technique.

**Fig. 1:**
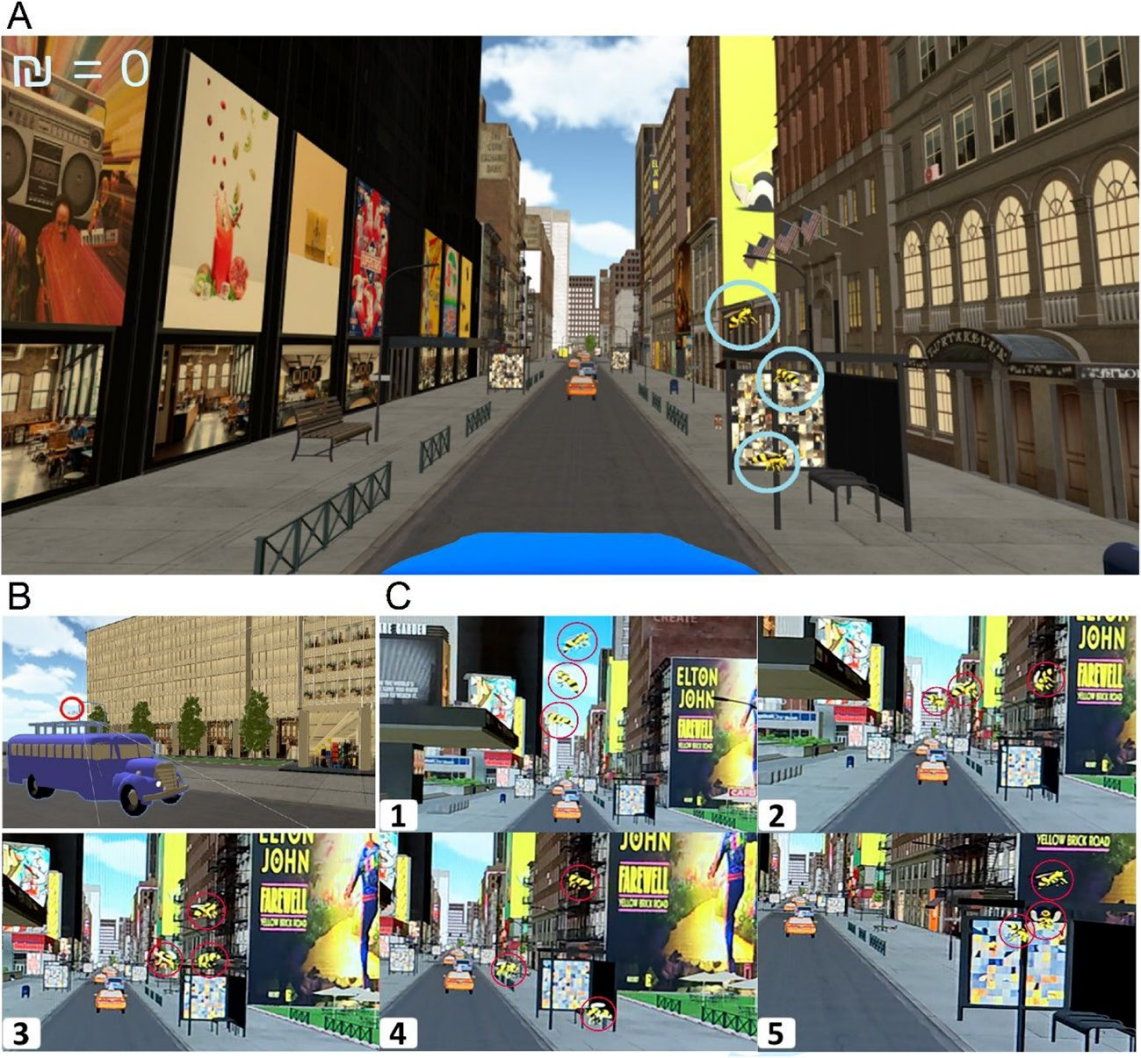
The VRIB environment. **A** A snapshot of the VR environment at a specific moment in the trial. Three bees (marked in light blue circles) fly around in random motion within a horizontally moving sphere, such that they overlay each bus stop on the side of the road. The bus stops depict either an intact or scrambled version of the target stimulus (here, scrambled images are presented due to copyright limitations on the IAPS images we used in the study). During each trial, the participants ride down the road; in the IB phase, their task is to follow the target bee. In the attended phase, their task is to gaze at the bus stops. In both conditions, subjective and objective measures of awareness of the critical stimulus are presented at the end of the trial. At the top left side, participants see their monetary gain (the symbol ₪ stands for NIS [new Israeli shekel], the currency in Israel). **B** A view of the bus on which participants’ point of view was located. The VR camera, through which the participants see their environment, is placed above the top railing of the bus (a white frame above the railing, marked in red). The bus itself was located at the center of a narrow one-lane road. During a trial, the bus was traveling down the urban road. **C** A trial sequence, zoomed in on the bee cluster for illustration purposes. Five frames (numbered 1–5), during which the bus progresses towards a bus stop (right) while the bees (circled in red) fly in its area. For a video demonstration see Supplementary Video

In our virtual reality IB paradigm (henceforth, VRIB), participants are immersed in an urban virtual environment, rich with detail (e.g., cars, taxis, billboards, storefronts; Fig. 1 and Supplementary Video). They are riding a bus in the city, while a group of three bees is flying around in front of them. Meanwhile, a stimulus image appears on top of bus stops located at both sides of the road; three bus stops present intact instances of the stimulus, and the other seven present the scrambled version of that stimulus. A trial ends once they have passed across ten bus stops. The experiment is divided into two phases: in the first phase (*IB phase*; 40 trials), the task is to follow a single target bee out of the three bees. At the end of each trial, participants are asked to select the target bee for monetary reward/punishment (for correct/incorrect responses, respectively). Then, participants’ awareness of the stimulus image is measured, both subjectively and objectively (see Method for further details). Participants receive no feedback about their responses to the awareness probes, and are instructed to maximize their monetary gain in the bee task. In the second phase (*attended phase*; ten trials), they are presented with playbacks of selected trials they had previously played, but here they are asked to ignore the bee task and focus on the bus stops (containing the stimulus).

Given the novelty of this paradigm, we first ran two exploratory experiments on independent samples (*N* = 20 in each), to validate it. Then, we conducted a third, preregistered experiment (*N* = 20) to confirm the robustness and reproducibility of the results. In all three experiments, the VRIB paradigm was found to be highly effective in inducing strong and recurring IB of repeatedly presented salient stimuli, despite trial-by-trial probing of awareness. Importantly, we further show that participants gazed at the critical stimuli for seconds, yet still failed to consciously perceive them in most trials. Thus, this method is highly effective in making people repeatedly fail to see a stimulus despite being asked about it directly.

## Method

### Participants

Sixty participants were included in this study overall. Twenty participants (14 female, aged 20–34, *M* = 25.77, *SD* = 3.96) were run in the preregistered experiment. One additional participant did not complete the preregistered experiment and was therefore excluded from further analysis, in line with the preregistered criteria. Similarly, 20 participants were run in each of the two exploratory experiments (Exploratory 1: 17 female, aged 20–29, *M* = 24.29, *SD* = 2.32; Exploratory 2: 16 female, aged 19.5–30.8, *M* = 25.21, *SD* = 3.19). All participants had normal or corrected-to-normal vision. Informed consent was obtained prior to each experimental session, and participants were compensated for their participation (regardless of the monetary reward they received based on their performance in the task). The sample size of the preregistered experiment was predetermined based on power analysis: We defined the effect of the manipulation (IB vs. attended phase) on the subjective report as the effect of interest (see Analysis). We collapsed both pilot experiments (*N* = 40) and found that the experimental phase affected visibility (β = −7.10, *p* < 0.001). We simulated new data and tested how many participants were required to reach 95% statistical power to find a similar effect. As the effect size was large, we found that *N* = 5 already provided a significant result with 95% power. Therefore, the sample size was determined to be identical to the ones used in the previous pilots (*N* = 20).

### Stimuli

#### Virtual environment

The virtual environment (Fig. 1A) was based on a 3D model of a city (source: turbosquid.com). The original model was heavily modified to create a controlled yet visually rich environment. The city’s roads and sidewalks were altered to create a single-lane, narrow street, which served as the experimental environment. The city’s buildings were edited, and additional objects were added (e.g., benches, billboards, logos, posters, storefronts, cars, cats and dogs, taken either from turbosquid.com or from Unsplash.com). A bank of 65 additional images was created using free online resources (mostly from Unsplash.com), depicting content that can be placed on billboards, posters, and storefronts, to mimic a typical street. In each trial, these images were randomly placed to diversify the street experience between trials and prevent participants from learning the path.

To present the target stimuli, ten bus stops were added on both sides of the road. The size of the bus stops was set to match the proportion of the urban environment (which was true to realistic size with respect to the participant, spaced about 40 Unity units from one another, corresponding to 40 “virtual meters”). Two additional bus stops with random images from the abovementioned content bank were presented in the street horizon (a part of the environment which participants never reached, as the bus stopped earlier), to substantiate the realness of the environment and its continuity.

In this urban environment, the participant’s point of view was located on top of a bus driving in the middle of the road (Fig. 1B). The bus was also based on a 3D model (source: tubrosquid.com). Its size was modified to fit the city, and it was colored blue (RGB: 69, 75, 211). The top part of the bus contained a railing, behind which the participant’s point of view was located (as if the participant were riding on the top deck of the bus). The bus speed was 30 Unity units per second and was kept constant across trials. Participants viewed the environment via an HTC VIVE Pro Eye headset and interacted with it using the HTC VIVE Pro Eye controller, which they held in their dominant hand.

#### Target stimuli and distractors

The target stimuli were 40 images selected from the International Affective Picture System database (IAPS: Lang et al., 2008), so that 20 were aversive and 20 were neutral. The IAPS was chosen to demonstrate how the paradigm could be potentially used to study unconscious processing, by comparing two classes of stimuli (Mudrik & Deouell, 2022). We specifically focused on aversive and neutral stimuli because this contrast was previously found to elicit strong effects (Bishop et al., 2004; Olofsson et al., 2008; Pessoa, 2005, 2008). However, the goal of the current study was to test the effectiveness of the attentional manipulation, rather than to test emotional processing without awareness. Therefore, comparing responses between neutral and aversive stimuli in this work was strictly exploratory (see preregistration: osf.io/648bp/), and more relevant for future work.

To select the images, we first defined negative and neutral images based on the original IAPS arousal and valence ratings. Aversive stimuli (*N* = 49) were defined as images with a mean arousal score equal to or larger than 6 (*M* = 6.52, *SD* = 0.34) and mean valence equal to or smaller than 4 (*M* = 2.54, *SD* = 0.66; 4 was chosen as a threshold because a score of 5 was prevalent in the positive stimulus set). Neutral stimuli (*N* = 58) were images with a mean arousal score equal to or lower than 5 (*M* = 4.03, *SD* = 0.60) and a valence score between 4 and 7 (*M* = 6.02, *SD* = 1.01). Then, images were manually excluded based on the following criteria: images that were monochromatic in color, images that we judged to be aversive/neutral in the neutral/aversive classes, respectively (e.g., an image of missiles in the neutral group), and images with similar semantic content (e.g., if there were two snake images, only one of them was included).

An online pilot was performed to validate the selected aversive and neutral stimuli. Fifty participants were recruited from the Prolific online platform in exchange for payment (7.5£/hour). During the experiment, participants were presented with all 40 stimulus images for unlimited time and were asked to rate the valence and arousal of each image on a five-point scale. The scale was the verbal parallel of the Self-Assessment Manikin rating of valence and arousal (SAM: Lang et al., 2008), with 1 and 5 representing unhappy and happy, respectively, in the valence scale, and calm and excited in the arousal scale, respectively. A Mann-Whitney *U*-test (*U* = 398.0, *p* < 0.001, rank biserial correlation = 0.99) confirmed that the valence ratings of neutral stimuli (*M* = 3.398, *SD* = 0.753) were higher than those of aversive stimuli (*M* = 1.328, *SD* = 0.273). We also found higher arousal for the aversive group (*M* = 3.244, *SD* = 0.129) than for the neutral group (*M* = 2.516, *SD* = 0.289; *U* = 0.00, *p* < 0.001, rank biserial correlation = −1.00). Scrambled versions of these images were prepared by splitting each image to 154 squares and shuffling them randomly using an in-house Python code. This allowed us to keep most of the low-level features of the images (e.g., luminance, saturation) while diminishing any semantic coherence.

Additional images were used for the objective awareness measure. Quadruples containing a target image and three distractors were manually selected from the IAPS database, such that none of the distractors was a target image. Each quadruplet included one distractor from the same valence group as the target stimulus (i.e., negative IAPS images for aversive stimuli, and either negative or positive IAPS images for neutral stimuli), while two distractors were from the opposite valence group. Thus, each quadruplet included two aversive stimuli and two neutral ones. Notably, the valence and arousal thresholds were not applied when selecting distractors, as there were not enough images in the IAPS emotion dataset that met these criteria. Distractors were chosen so they would be chromatically close to the target image, but not identical to the target stimulus content (e.g., for a snake target stimulus, there was no snake distractor).

During each trial, a single target image appeared on the ten experimental bus stops: its intact version appeared on three randomly selected bus stops, and its scrambled version appeared on the other seven. This was done to allow jittering of the location of the meaningful stimuli in each trial, without introducing other meaningful images that might evoke conflicting processes. Notably, including scrambled stimuli also allows one to probe semantic processing (by comparing responses to meaningful vs. meaningless stimuli with similar low-level features).

#### Task stimuli

The bee stimuli were created from an online asset of a bee-shaped object (source: turbosquid.com), which was then colored in black and yellow stripes (yellow RGB: 255, 204, 53). These bees moved in the environment in a way that made it seem like they were flying in front of the participant (for more detail about their speed, see Procedure). To ensure that participants’ gaze would be directed towards the target stimulus, the movement path of the three bees was manipulated so that they would fly over the stimulus. To that end, the bees were placed within an invisible sphere, and their motion was limited by its boundaries. The sphere moved horizontally between bus stops, such that the bees overlapped with all instances of the stimulus. The edges of the sphere’s movement were defined as ±20° visual angle (with 0° being right across from where the participant was located). The bees’ motion was programmed such that at any given moment, each bee flew towards a randomly chosen point on the perimeter of the invisible sphere. Once it got close enough to the selected point, a new point was generated, and so on. To make these transitions look more realistic, the motion was smoothed using a linear interpolation between locations.

#### Music

All the experimental trials (in both the IB and the attended phases) were accompanied by music, to enhance participants’ engagement. Twelve musical segments were created by snipping instrumental pieces from Bensound, a copyright-free music platform. They were played according to the alphabetical order of their names (counterbalanced between ascending and descending orders across participants).

### Apparatus

The paradigm was developed in Unity (version 2019.4.17f1) and designed to run with an HTC VIVE Pro Eye virtual reality headset. The experiment was run on a computer with an Intel Core i9 processor with an RTX 3090 GAMING OC graphics card and a Microsoft Windows 10 operating system. Participants’ responses were recorded using the right HTC VIVE Pro Eye controller (which participants held in their dominant hand throughout the experiment).

Binocular gaze measurements were recorded using the eye-tracking technology embedded in the headset. The gaze data were recorded and logged using Unity’s collider features for marking the objects of interest—the ten bus stops on which the target stimulus was presented. Every time participants’ gaze was directed at one of the colliders, the timepoints marking the start and end of that gaze were saved. Other than these timestamps, no other type of data were extracted from the eye tracker.

### Procedure

The experiment consisted of an *IB phase*, followed by an *attended phase*. Prior to wearing the headset, participants were briefed about the VR headset and controller, and about the eye-tracking calibration and validation procedures. Then they were presented with pictures of the virtual environment, the bees, and the questions to be presented at the end of each trial, to familiarize them with the task. They were instructed that they would win or lose money based only on selection of the right bee, and to maximize their gain and avoid distractions as much as possible. Once participants wore the headset, the built-in eye-tracker calibration was performed, followed by a validation procedure that is embedded in the VRIB platform.

Then the IB phase (40 trials) began. There, each trial started with the bus standing still and the bees appearing motionless, ahead of the participant, with one of the bees marked as the target (to be tracked by the participant) using a translucent light-green sphere surrounding it (RGB: 15, 191, 0; alpha: 98). For the first three seconds, the bees and bus were still. Then, the bus started to move down the road and the bees started to fly randomly within the sphere, with the sphere moving towards the nearest bus stop. After an additional five seconds, the marking of the target bee disappeared (the target bee was marked for a total of eight seconds). Since tracking the bees is challenging, we wanted to prevent a situation where participants accidentally lost eye contact with the target bee and stopped being attentionally engaged by the main task. To that end, participants were told that they could ask for clues whenever they lost track of the target bee. A clue was requested by pressing the controller’s trigger button, which marked the target bee in green for three seconds. Clues cost money (0.5 NIS at the beginning of each trial, doubling to 1 NIS towards the end to discourage a strategy of not following the bee throughout the trial and asking for a clue right before the probe), and there was no limitation on the number of clues participants could ask for during a trial (i.e., participants could have a negative balance if they asked for clues worth more than what they had gained; if the IB phase ended with a “debt,” participants were reimbursed only for their participation, and did not receive extra monetary reward for their performance).

After 1:03 minutes, when the bus ride ended, the cluster of bees returned to the center and stopped moving. The participants were then asked to select the target bee that was marked at the beginning of the trial using the VIVE controller. Upon correctly choosing the target bee, participants gained money (2 NIS); alternatively, if a non-target bee was selected, participants lost money (2 NIS; the IB phase started with zero NIS). Feedback on performance on the bee task was given immediately, alongside the updated total sum of the money they gained, which appeared at the top-left corner of the display (see Fig. 1A). To assess whether participants were aware of the images on the bus stops, we used two awareness measures. Firstly, participants were asked to rate stimulus visibility on the Perceptual Awareness Scale (PAS, a subjective measure of awareness: Ramsøy & Overgaard, 2004), and then they were asked to select the stimulus image from an array of four images (an objective measure: Jakel & Wichmann, 2006). None of the questions at the end of the trial were limited in time. Similarly, a trial began only once participants pressed a button to indicate they were ready.

To maintain engagement, the speed of the bees was modulated based on participants’ success in the main task: When they were correct in selecting the target bee, the speed on the following trial increased (by 0.1 Unity units per second), and vice versa when they were wrong. When participants were correct but asked for three or more clues, the speed in the subsequent trial did not change. Notably, participants’ responses to the awareness measures did not affect the bees’ speed or their monetary rewards. The experiment began with an initial bee speed of 1.0 Unity units per second, the minimum speed was 0.1, and the maximum speed was 3.0.

At the end of the IB phase, participants were informed about the final amount of money they had won, and the attended phase (ten trials) started. There, recordings of a subset of the trials from the IB phase were played back to the participant (such that this phase was uniquely generated per participant). This time, participants were asked to focus their attention on the bus stops and were accordingly not reimbursed for selecting the target bee. Other than the verbal instructions, the IB phase and the attentional phase were identical, including the movement patterns of the bees and all other experimental factors.

## Statistical analysis

Data preprocessing was performed in Python (Van Rossum & Drake, 2009). We modeled mixed-effects regression in R (R Core Team, 2022) using the lme4 package, and compared models using Bayes factors based on Bayesian information criterion (BIC) approximation (Wagenmakers, 2007), using the BayestestR package (Makowski et al., 2019). The rest of the statistical tests were executed in JASP (version 0.16.3.0; JASP Team, 2022). All following analyses were preregistered.

Our first aim was to test *whether the paradigm is effective in inducing multi-trial inattentional blindness*. For that, we first focused on *subjective ratings of awareness*, as indicated by participants’ PAS responses. If the IB phase effectively manipulated visibility, stimuli were expected to be reported as less visible than in the attended phase. Furthermore, to address the “inattentional amnesia” argument, we tested whether visibility ratings depended on the time interval between the last intact stimulus appearance and the PAS prompt. We reasoned that if stimuli became less visible the longer the lag between the stimulus and the prompt, this would strengthen claims that the observed results reflect a memory failure rather than a perceptual one. Accordingly, we used an ordinal regression predicting the visibility rating on the PAS as a function of Condition (IB/attended phase) and SOA_S_ (stimulus onset asynchrony [SOA] subjective; the time between the last intact stimulus and the PAS prompt) as fixed effects, and Subject (participant) as a random effect:

$$H1:PAS \sim Condition+{SOA}_{s}+\left(Condition+{SOA}_{s} \right| Subject)$$

To assess the contribution of each of the independent variables, we compared model H1 to the two following null models:

$$\begin{array}{c}{H0}_{1}:PAS \sim {SOA}_{s}+\left(Condition+{SOA}_{s} \right| Subject)\\ {H0}_{2}:PAS \sim Condition+\left(Condition+{SOA}_{s} \right| Subject)\end{array}$$

We next focused on *objective measures of awareness*, asking *whether performance in the objective task differed from chance* when stimuli were reported invisible (according to the PAS rating). Following many studies in the field (for a review, see Mudrik & Deouell, 2022; Seth et al., 2008), we hypothesized that chance performance in discriminating subjectively invisible stimuli would validate subjective reports and reinforce the claim that stimuli were not consciously perceived. Therefore, we performed a Bayesian one-sample *t*-test against chance-level performance (25%) using the average accuracy per participant in visibility 1 trials (where the participant rated visibility as “1” in the PAS probe, indicating not seeing the stimulus image at all). Similarly to the subjective measure, we also tested whether performance in the objective task depended on the time interval between the last intact stimulus appearance and the objective question. We used a logistic regression predicting correct responses as a function of SOA_O_ (SOA objective; the time between the last presentation of an intact critical stimulus and the objective question) as a fixed effect, and Subject (participant) as a random effect:

$$H1:Correct \sim {SOA}_{o}+\left({SOA}_{o} \right| Subject)$$

To assess the contribution of the independent variable, we compared model H1 to the following null model:

$$H0:Correct \sim 1+\left({SOA}_{O} \right| Subject).$$

Our second aim was to *examine gaze behavior, to test whether participants indeed directed their gaze towards the critical stimuli*. This was done to demonstrate that the reported invisibility of the stimulus was not due simply to not looking in the general direction of the bus stop (i.e., that participants “looked but failed to see” rather than not gazing in that direction; Langham et al., 2002; White & Caird, 2010). We examined the average gaze duration on all presentations of the intact critical stimulus in each visibility 1 trial by plotting the trial data and performing a Bayesian one-sample *t*-test against zero.

In addition to the abovementioned preregistered analyses, two classes of exploratory analyses were conducted: the first class focused on better characterizing behavior in the VR environment. We *tested whether participants indeed complied with the task instructions*, by performing a Bayesian one-sample *t*-test to examine whether performance in the bee task was different from chance level (33%). Then, we examined *whether participants’ engagement with the game changed with time*, as indicated by the number of requested clues during each trial. Clue requests indicate that participants were still motivated and tried to successfully complete the task, as they were willing to pay to get a hint. To test whether the number of clues participants requested during a trial was predicted by the trial number, we used linear regression modeling the number of clues requested during a trial as a function of the trial number:

$$H1:Clues Taken \sim Trial Number+\left(Trial Number \right| Subject)$$

To assess the contribution of the independent variable, we compared model H1 to the following null model:

$$H0:Clues Taken \sim 1+\left(Trial Number \right| Subject)$$

As IB has not been previously repeated for multiple trials when measuring awareness, the VRIB uniquely allows us to examine how awareness reports in an IB paradigm change with time. Accordingly, we tested *whether subjective reports and objective performance changed as the experiment progressed*. To that end, we compared a linear regression modeling PAS as a function of trial number:

$$H1:PAS \sim Trial Number+\left(Trial Number \right| Subject)$$

to the following null model:

$$H0:PAS \sim 1+\left(Trial Number \right| Subject)$$

Similarly, we used a logistic regression modeling correct responses as a function of trial number:

$$H1:Correct \sim Trial Number+\left(Trial Number \right| Subject)$$

compared to the following null:

$$H0:Correct \sim 1+\left(Trial Number \right| Subject)$$

The second class of exploratory analyses focused on potential markers of unconscious processing of the stimuli, using eye-tracking data. We examined gaze duration patterns to see *whether there were differences between intact and scrambled instances of a stimulus*. Such differences imply that the meaning of the stimulus was processed to some extent. Thus, we performed *t*-tests comparing gaze durations towards intact and scrambled instances, separately for the IB phase and the attended phase.

Lastly, as our stimulus database comprised aversive and neutral stimuli, we explored *whether there were differences in gaze patterns or behavior between aversive and neutral stimuli,* with the same rationale that such differences would imply that their content was processed. We used *t*-tests to compare gaze duration towards intact instances of the stimulus between the aversive and neutral groups, separately for visibility 1 trials (where participants reported the stimulus as unseen) and visibility 4 trials (where participants reported the stimulus as clearly visible).

## Results

Below, we report descriptive and inferential statistics, following the analyses described in the Statistical analysis section. We include both preregistered and exploratory analyses under each section, differentiating between them. For each analysis, we first report the results of the preregistered experiment, and then of the two exploratory experiments. The only exceptions are the analyses containing SOA, which were performed only on the preregistered sample (as information about the SOA was only collected on that sample).

## Did the task effectively induce multi-trial inattentional blindness?

### Subjective rating of awareness

**Fig. 2:**
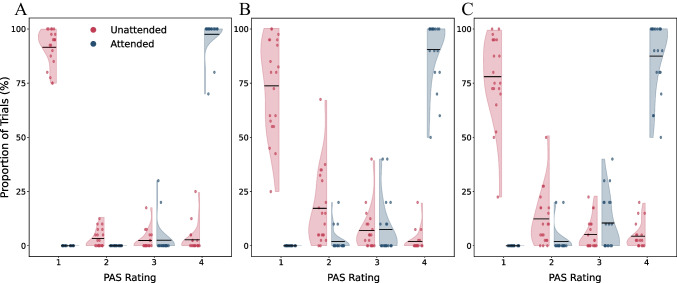
Proportion of trials per PAS rating, in the attended (blues) and IB (reds) phases. **A** The preregistered sample. **B** Exploratory Experiment 1. **C** Exploratory Experiment 2. In all three panels, the *X* axis is the PAS rating, and the *Y* axis is the proportion of trials where rated as such

Despite being presented with a similar display over multiple times and being repeatedly questioned about the content of their perception, participants rated stimuli as mostly unseen in the IB phase (PAS 1, corresponding to not having any experience of the stimulus: *M* = 91.5%, *SD* = 8.64). On the contrary, in the attended phase (ten trials), stimuli were easily seen (PAS 4, corresponding to having a clear experience of the stimulus: *M* = 97.5%, *SD* = 7.86; Fig. 2A).

Similar results were obtained in the two exploratory experiments (PAS 1 in the IB phase: Exploratory E1: *M* = 73.75%, *SD* = 22.31; Exploratory E2: *M* = 78%, *SD* = 19.93; PAS 4 in the attended phase: Exploratory E1: *M* = 90.5%, *SD* = 15.04; Exploratory E2: *M* = 87.5%, *SD* = 16.18; Fig. 2B and C). Modeling the PAS scores revealed that in the IB phase, visibility scores were lower than in the attended phase (β = −13.26, *p* < 0.001; BF_10_ = 4.76 × 10^4^). This was again found for the two exploratory studies, where we ran the model without SOA_S_, which were not recorded in those experiments (Exploratory E1: β = −7.84, *p* < 0.001, BF_10_ = 5.80 × 10^7^; Exploratory E2: β = −6.50, *p* < 0.001, BF_10_ = 8.23 × 10^8^).

### Performance in the objective task

Performance in visibility 1 trials was not different from chance level (*M* = 23.42%, *SD* = 6.37, *t*-test against 25%: *t*(19) = −1.11, *p* = 0.28, Cohen’s *d* = −0.25, 95% CI = [−0.69, 0.20], BF_10_ = 0.40). The same result was found in the two exploratory experiments we conducted (Exploratory E1: *M* = 28.22%, *SD* = 13.56, *t*-test against 25%: *t*(19) = 1.06, *p* = 0.30, Cohen’s *d* = 0.24, 95% CI = [−0.21, 0.68], BF_10_ = 0.38; Exploratory E2: *M* = 23.30%, *SD* = 9.23, *t-*test against 25%: *t*(19) = −0.83, *p* = 0.42, Cohen’s *d* = −0.18, 95% CI = [−0.62, 0.26], BF_10_ = 0.31). The objective measure thus validates the subjective ratings, providing strong evidence that the VRIB paradigm repeatedly suppressed the stimuli from awareness.

### Can the results be explained by inattentional amnesia?

**Fig. 3:**
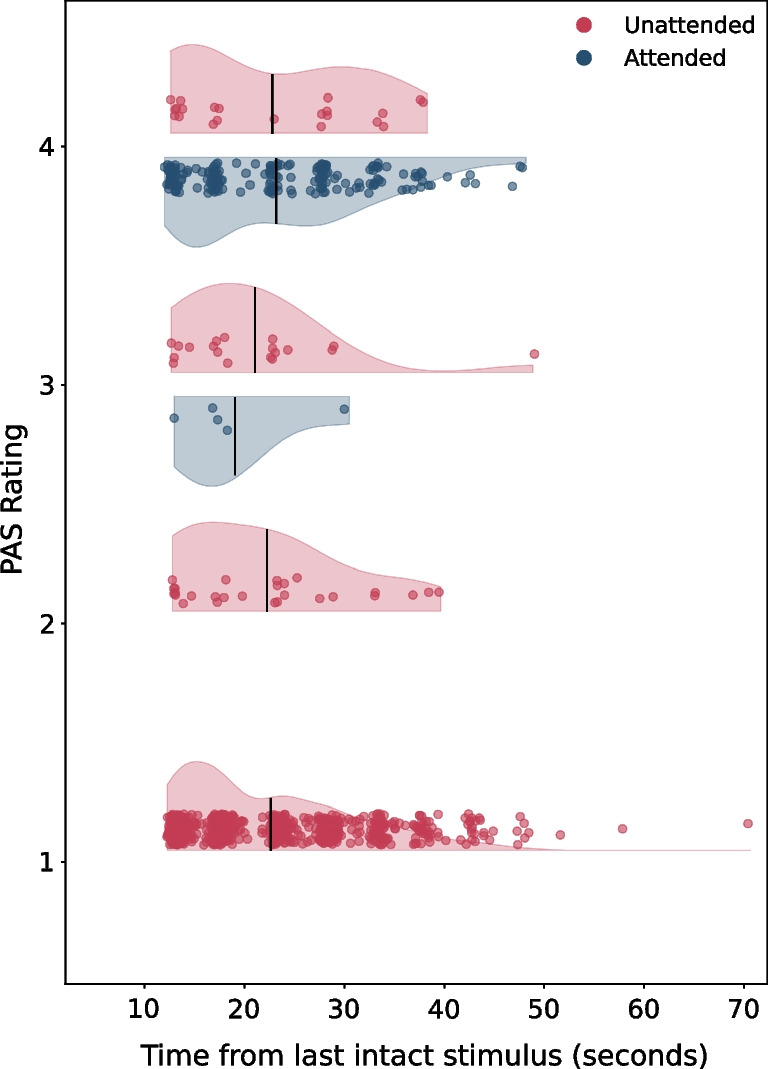
Time between the last intact instance of a critical stimulus in a trial, and the trial’s PAS rating (SOA_s_) in the attended (blue) and IB (red) phases. *X* axis: time (in seconds) between the participant passing across the last intact instance of a stimulus, and the PAS prompt. *Y* axis: PAS. Each point represents the time between the last intact instance of the stimulus and PAS, for a certain PAS rating in a single trial. Black lines denote the average time for each visibility rating. The data presented here are from the preregistered experiment

According to the “inattentional amnesia” account, participants experience the stimuli but forget them. If so, one would expect lower ratings the greater the delay between the stimulus and the probe, as visual memory decays with time (Phillips & Baddeley, 1971; Posner & Keele, 1967). We accordingly asked whether these measures were modulated by the time that had passed since the last appearance of the critical stimulus. We found no relation between the elapsed time and the subjective awareness prompt (β = −0.01, *p* = 0.63; BF_10_ = 0.04; Fig. 3), mitigating concerns that participants did consciously perceive the stimulus but had forgotten when prompted. Similarly, no effect was found for the objective task (β = −0.01, *p* = 0.17; BF_10_ = 0.08). Thus, the concern that these measures reflect memory rather than conscious perception seems less plausible (notably, this analysis was not conducted on the data from the exploratory studies, as SOA was not recorded there).

## Did participants gaze at the critical stimuli?

**Fig. 4:**
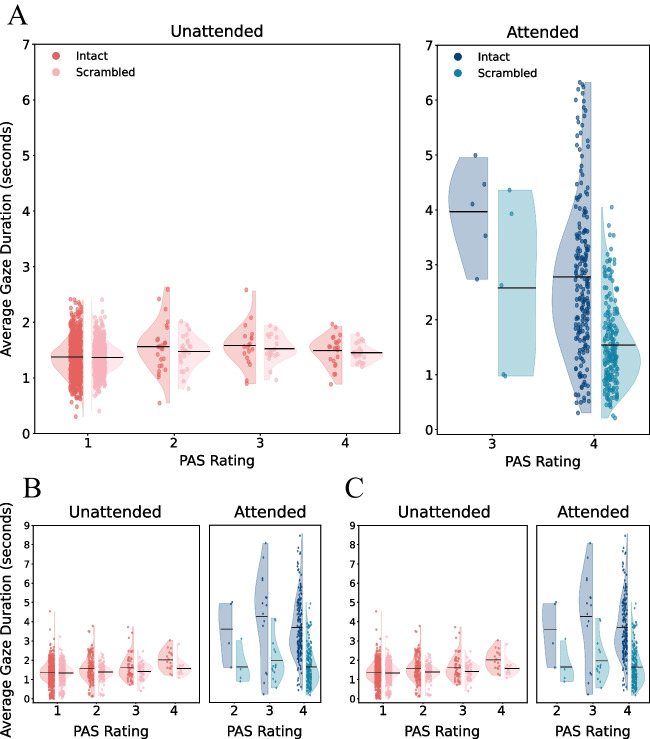
Average gaze duration (in seconds) towards the critical stimulus image during unattended trials (left) and attended trials (right), per PAS rating. The data are presented separately for intact (left-oriented violins: red [unattended] and blue [attended]) and scrambled (right-oriented violins: pink [unattended] and turquoise [attended]) versions of the stimulus. Each dot represents the average gaze duration across bus stops, for a certain PAS rating, within a single trial (i.e., not aggregated per participant). Horizontal lines depict the average value for each distribution. **A** Data from the preregistered experiment. **B** Data from the first exploratory experiment. **C** Data from the second exploratory experiment

We asked whether participants’ gaze was directed towards the target stimuli. Otherwise, their report of not seeing the stimuli would not stem from the attentional manipulation, but simply because they did not look at them. The results suggest otherwise: the average gaze duration on intact stimuli during visibility 1 trials was larger than zero, and relatively long (Fig. 4; *M* = 1.37 sec, *SD* = 0.34; *t*(722) = 108.76, *p* < 0.001, Cohen’s *d* = 4.04, 95% CI = [3.82, 4.26], BF_10_ = ∞). Again, this was also evident in the exploratory experiments (Exploratory E1: *M* = 1.37, *SD* = 0.54, *t*(587) = 61.77, *p* < 0.001, Cohen’s *d* = 2.55, 95% CI = [2.38, 2.71], BF_10_ = 6.42 × 10^254^; Exploratory E2: *M* = 1.40, *SD* = 0.38, *t*(621) = 91.97, *p* < 0.001, Cohen’s *d* = 3.69, 95% CI = [3.47, 3.91], BF_10_>10^5^). And, notably, this was true for all individual trials, as can also be seen in Fig. 4.

## Were participants complying with the VRIB task?

Overall, participants were immersed in the platform, and none reported having motion sickness during the experiment. Their performance on the main task (i.e., the bee task during the IB phase) was relatively high (*M* = 78%, *SD* = 9.70, *t*-test against chance at 33%: *t*(19) = 20.85, *p* < 0.001, Cohen’s *d* = 4.66, 95% CI = [3.12, 6.19], BF_10_ = 3.63 × 10^11^). Similar performance was observed in both exploratory experiments (Exploratory E1: *M* = 76.2%, *SD* = 8.00, *t*(19) = 24.27, Cohen’s *d* = 5.43, *p* < 0.001, 95% CI = [3.66, 7.19], BF_10_ = 5.04 × 10^12^; Exploratory E2: *M* = 77%, *SD* = 11.5, *t*(19) = 17.13, Cohen’s *d* = 3.83, *p* < 0.001, 95% CI = [2.54, 5.11], BF_10_ = 1.27 × 10^10^). However, the task was not easy for the participants, as reflected by their average number of clue requests per trial (preregistered experiment: *M* = 1.35, *SD* = 0.88; Exploratory E1: *M* = 1.08, *SD* = 0.61; Exploratory E2: *M* = 1.58, *SD* = 1.10).

### Changes in task behavior over time

**Fig. 5:**
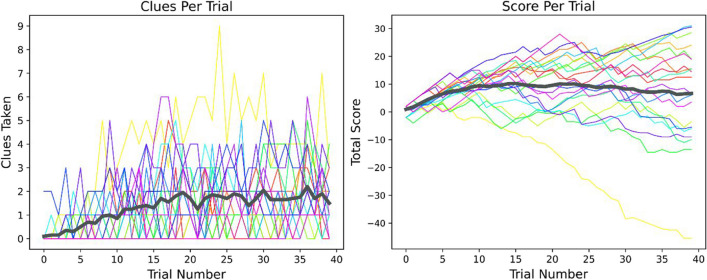
VRIB platform IB phase task performance. **Left panel**: The number of clues requested during a trial. *X* axis: trial number, *Y* axis: the number of clues. **Right panel**: The total score accumulated by the participants at the end of each trial. *X* axis: trial number, *Y* axis: the accumulated score. In both panels, the thick dark gray line depicts the average across participants, and individual lines represent individual participants. The data in this figure depict the preregistered sample

We further asked whether the trial number predicted the number of clues participants requested during the trial. We found a significant relationship between the trial number and the number of clues taken (β = 0.04, *p* < 0.001; BF_10_ = 2.59 × 10^3^; Fig. 5, left panel), meaning that, as the IB phase progressed, participants requested more clues to help them complete the bee task successfully. Similar results were present in both exploratory experiments (Exploratory E1: β = 0.03, *p* < 0.001; BF_10_ = 1.89 × 10^3^, Exploratory E2: β = 0.04, *p* = 0.001; BF_10_ = 7.96).

This might be explained either by the adaptive difficulty of the task (as the speed of the bees increased/decreased following a correct/incorrect response), or by fatigue due to the prolonged engagement in the task. Importantly, this does not represent reduced engagement with the task, or reduced motivation to succeed: after an initial overshoot, the total monetary gain of participants was generally stable (though note that while some participants improved during the game, others deteriorated, as is evidenced by the increased variability; Fig. 5, right panel). This suggests that participants kept their initial motivation to succeed in the task, and that the dynamic speed of the bees was indeed effective in keeping participants challenged and engaged.

### Changes in awareness measures over time

To assess the effectiveness of the paradigm in inducing IB over time, an additional exploratory analysis tested whether the subjective or objective scores changed as a function of trial number (i.e., the time that had passed during the experiment). Modeling PAS ratings by trial number revealed that as the experiments progressed, stimulus visibility increased slightly, as might be expected (β = 0.22, *p* < 0.001; BF_10_ = 5.95 × 10^25^). Similar results were achieved for the two exploratory studies (Exploratory E1: β = 0.16, *p* < 0.001, BF_10_ = 4.16 × 10^17^; Exploratory E2: β = 0.16, *p* < 0.001, BF_10_ = 3.30 × 10^5^). In the objective task, the trend of improved performance with trial number was evident, but it was only confirmed by the Bayesian analysis and not the frequentist one (β = 0.41, *p* = 0.108, BF_10_ = 3.64 × 10^8^). In the exploratory studies, performance did improve as the experiment progressed (Exploratory 1: β = 1.24, *p* < 0.001, BF_10_ = 5.40 × 10^5^; Exploratory 2: β = 0.07, *p* < 0.001, BF_10_ = 3.22 × 10^5^).

## Did gaze patterns reveal any sign of content processing without awareness?

### Intact versus scrambled stimuli

**Fig. 6:**
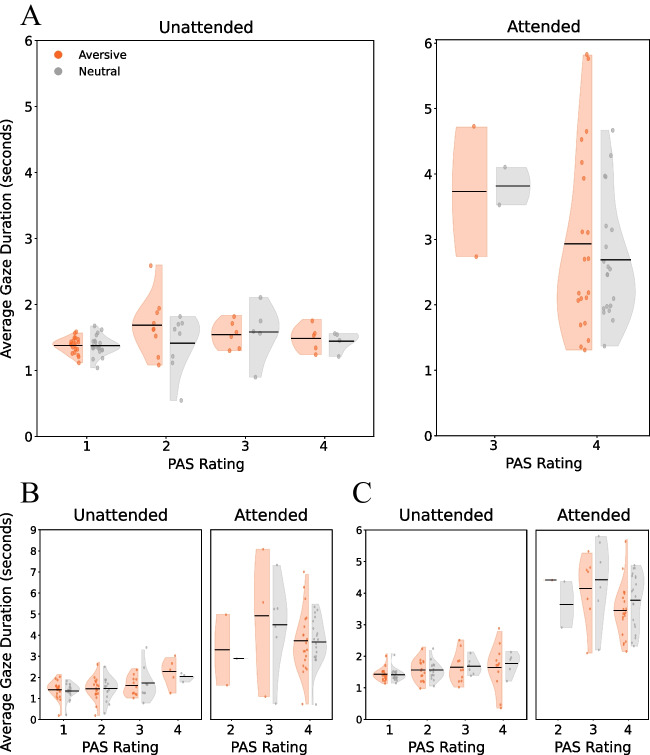
Average gaze duration per participant (in seconds) towards the intact instances of the critical stimulus image during unattended trials (left) and attended trials (right), per PAS rating. The data is plotted separately for aversive (left-oriented violins; orange) and neutral (right-oriented violins; gray) stimuli. Each dot represents a single participant’s average gaze duration across all intact bus stops during all trials with a certain PAS rating. Horizontal lines depict the average value for each distribution. **A** Data from the preregistered experiment. **B** Data from the first exploratory experiment. **C** Data from the second exploratory experiment

Further exploratory analyses revealed that in the attended phase, participants looked more at intact instances of the target stimulus (*M* = 2.81, *SD* = 1.11) than at scrambled ones (*M* = 1.56, *SD* = 0.52; *t*(19) = 6.24, *p* < 0.001, Cohen’s *d* = 1.40, 95% CI = [0.76, 2.01], BF_10_ = 3868.57). However, during the IB phase, no difference was found between gaze duration towards intact (*M* = 1.39, *SD* = 0.13) and scrambled instances of the stimulus (*M* = 1.37, *SD* = 0.12; *t*(19) = 1.20, *p* = 0.246, Cohen’s *d* = 0.27, 95% CI = [−0.18, 0.71], BF_10_ = 0.43; Fig. 6A). Similar results were obtained in the exploratory samples (Exploratory E1: IB phase: intact *M* = 1.44, *SD* = 0.39, scrambled *M* = 1.38, *SD* = 0.33, *t*(19) = 1.59, *p* = 0.13, Cohen’s *d* = 0.36, 95% CI = [−0.10, 0.80], BF_10_ = 0.68, Attended phase: intact *M* = 3.57, *SD* = 1.14, scrambled *M* = 1.70, *SD* = 0.77, *t*(19) = 10.46, *p* < 0.001, Cohen’s *d* = 2.34, 95% CI = [1.47, 3.19], BF_10_ = 4.47 × 10^6^; Exploratory E2: IB phase: intact *M* = 1.46, *SD* = 0.04, scrambled *M* = 1.44, *SD* = 0.15, *t*(19) = 0.77, *p* = 0.45, Cohen’s *d* = 0.17, 95% CI = [−0.27, 0.61], BF_10_ = 0.30, Attended phase: intact: *M* = 3.73, *SD* = 0.77, scrambled *M* = 1.65, SD = 0.44, *t*(19) = 10.80, *p* < 0.001, Cohen’s *d* = 2.42, 95% CI = [1.53, 3.29], BF_10_ = 7.35 × 10^6^; Fig. 6B and C).

## Did gaze patterns reveal any sign of emotional processing without awareness?

With respect to gaze patterns, during visibility 1 trials (which occurred only during the IB phase; Fig. 6A), the duration of gaze towards the intact instances of the stimulus did not change between aversive and neutral stimuli (aversive: *M* = 1.38, *SD* = 0.12; neutral: *M* = 1.38, *SD* = 0.15; *t*(19) = 0.15, *p* = 0.88, Cohen’s *d* = 0.03, 95% CI = [−0.41, 0.47]; BF_10_ = 0.23). Similar results were achieved in the exploratory samples (Exploratory E1: aversive *M* = 1.41, *SD* = 0.41, neutral *M* = 1.36, *SD* = 0.35, *t*(19) = 1.31, *p* = 0.20, Cohen’s *d* = 0.29, 95% CI = [−0.16, 0.74], BF_10_ = 0.49; Exploratory E2: aversive *M* = 1.43, *SD* = 0.19, neutral *M* = 1.42, *SD* = 0.18, *t*(19) = 0.44, *p* = 0.67, Cohen’s *d* = 0.10, 95% CI = [−0.34, 0.54], BF_10_ = 0.25). However, a lack of significant difference was also evident during the attended phase, in trials reported to be fully visible (PAS 4; Fig. 6, right panel; aversive: *M* = 2.94, *SD* = 1.42; neutral: *M* = 2.69, *SD* = 0.92; *t*(19) = 1.56, *p* = 0.13, Cohen’s *d* = 0.35, 95% CI = [−0.11, 0.80]; BF_10_ = 0.66). Similar results were achieved in the exploratory samples (Exploratory E1: aversive *M* = 3.74, *SD* = 1.48, neutral *M* = 3.69, *SD* = 1.10, *t*(19) = 0.28, *p* = 0.78, Cohen’s *d* = 0.06, 95% CI = [−0.38, 0.50], BF_10_ = 0.24; Exploratory E2: aversive *M* = 3.47, *SD* = 0.84, neutral *M* = 3.79, *SD* = 0.88, *t*(19) = −1.51, *p* = 0.15, Cohen’s *d* = −0.34, 95% CI = [−0.78, 0.12], BF_10_ = 0.61). Therefore, both when reported seen and unseen, gaze patterns were not affected by stimulus valence (see Discussion below for possible reasons for this null result).

## Discussion

In a series of three experiments, we studied the effectiveness of the VRIB paradigm in inducing inattentional blindness in an ecologically valid environment. Our results show that even though participants knew that they would be asked about the unattended stimulus at the end of each trial, the VRIB paradigm still effectively induced IB over and over, across multiple trials. Furthermore, this blindness occurred even though the stimuli were viewed foveally for prolonged durations, and despite repeated exposure to the same visual display. Thus, the bee task effectively engaged top-down attention, yielding multi-trial IB where the stimulus was rendered unseen time and time again. The high visibility ratings and objective task performance in the attended condition further demonstrate that the same stimuli are easily seen in the same setup when attention is not captured by the bees. Taken together, our results demonstrate that the VRIB paradigm is a powerful tool for studying the extent of unconscious processing and its neural correlates in a more ecological manner.

This paradigm goes beyond previous IB experiments in several ways. First, our results demonstrate that IB can be repeatedly induced in the same participants for a considerable number of trials despite trial-by-trial reporting, which has typically been considered challenging (Hutchinson, 2019). With a sufficiently engaging task, IB can occur even when expectations about the unattended stimulus have already been formed. To our knowledge, this is the first time that IB has been repeated for more than a couple of trials within the same participant under these conditions.

Second, probing of awareness in our task was more rigorous than in previous IB experiments. The awareness probe in such studies is typically subjective (e.g., Cartwright-Finch & Lavie, 2007; Murphy & Greene, 2016; Pitts et al., 2012; Simons & Chabris, 1999; Ward & Scholl, 2015), and varies greatly between experiments (from a binary yes/no question, to answers whether there was “anything different,” to drawings and free reports). Even when including objective questions about the critical stimulus, there is again high variability between experiments, ranging between presenting forced-choice tasks (e.g., Koivisto et al., 2004; Thakral, 2011) and open-ended questions (e.g., Most et al., 2001), with no clear standard. Here, we combined a common subjective awareness metric (PAS: Ramsøy & Overgaard, 2004) with a four-alternative forced-choice task, having both objective and subjective awareness assessments at the end of each trial. Recent criticism suggested that in IB paradigms, participants can see more than what is reflected in their reports of noticing/not noticing (demonstrated in above-chance performance in a two-alternative forced-choice task; Nartker et al., 2021). However, in our work, performance on the objective task was not above chance, even though the objective probe was well expected and repeated many times. Notably, with gaze tracking, we demonstrate that the stimuli were repeatedly suppressed from awareness despite being viewed foveally.

Third, in IB paradigms, once participants are explicitly asked about the critical stimulus, the common assumption is that they would see it in subsequent trials (Hutchinson, 2019; Simons & Chabris, 1999). Thus, most experiments include only one trial (e.g., Simons & Schlosser, 2017), and those that have two trials change the unexpected event such that it would be different enough from the first stimulus, to increase the chances for IB (Murphy & Greene, 2016; Ward & Scholl, 2015). Here, we demonstrate that even when not changing the presentation of the critical stimulus (i.e., the stimulus was always an image, presented on top of bus stops, depicting some scene), and repeating it many more times (N = 40), people can still fail to consciously experience its content. Exploratory analyses revealed that visibility, as indicated by both the subjective and objective measures, increased with time, albeit to a relatively small extent. Notably, this trend is not unique to our manipulation, and might be attributed to practice effects (in masking: Dorais & Sagi, 1997; in crowding: Huckauf & Nazir, 2007).

Fourth, our results show that IB can also be induced in VR environments, where participants not only watch dynamic, complex scenes, but also interact with them in a more ecologically valid manner. This offers new possibilities for studying conscious versus unconscious processing, which are not available when using on-screen stimuli (e.g., having participants interact with an immersive environment). It also provides researchers with the opportunity to enrich the collected data, extending beyond what is commonly used in computer experiments (e.g., gait and motion: Palmisano et al., 2022; Scarfe & Glennerster, 2015). While a previous attempt to induce IB in VR did not replicate the gorilla effect (Schöne et al., 2021), our paradigm facilitated IB in VR, which generalizes the attentional effect.

On a more theoretical level, the high effectiveness of this paradigm further demonstrates the close ties between consciousness and attention, contributing to the discussion about their relationship (Aru & Bachmann, 2013; Cohen et al., 2012, 2016; Tsuchiya et al., 2012). Our findings suggest that the two are indeed tightly related: despite foveal viewing of the target stimuli, a potent enough manipulation of attention can very strongly hinder the conscious experience of the stimuli. This seems less compatible with studies showing that while engaged in a central task, participants can still succeed in reporting events presented in their visual periphery (e.g., Li et al., 2002; Reddy et al., 2006). In such dual-task paradigms, participants were able to detect target stimuli among distractors. However, it is possible that in these studies, some top-down attention was still allocated towards the peripheral task, as there was no procedure verifying that attention was indeed not allocated to the periphery. Here, the dynamic nature of the task, which modified itself based on participants’ performance to make sure they stay engaged, and the rich real-life environments were probably the critical factors driving the strong effect of attention on consciousness.

We suggest that this strong effect can be used to study the differences between conscious and unconscious processing. As a case in point, the eye tracking data in our experiment suggested a difference in the duration of gaze towards the intact and scrambled versions of the target stimulus during the attended phase (where stimuli were always consciously perceived) but not during the IB phase (where most of the stimuli were reported not to be consciously perceived). The positive result during the attended phase suggests that the meaning of the stimuli was processed, as participants differentiated semantically meaningful from meaningless instances. This result further demonstrates the effectiveness of the attentional manipulation: as VR headsets have fixed focal distances, focusing on a foreground object does not make the background display blurry. Therefore, when focusing on a foreground bee, it is unclear whether the target stimulus instances in the background have been the focus of attention or not (as the vergence of the eyes does not match their accommodation, a phenomenon called “vergence-accommodation conflict”; Cakmakci & Rolland, 2006). While future studies can collect gaze depth data to disambiguate instances of attention towards the foreground bees and the background images, the difference in gaze towards intact and scrambled stimuli in the attended phase (and the lack thereof during the IB phase) indicates that the focus of attention shifted between the phases.

More importantly, the lack of difference in gaze between intact and scrambled stimuli in the IB phase might suggest that there was no processing of scene meaning under inattentional blindness. This result is in line with previous studies reporting a failure to decipher scenes without awareness (e.g., Biderman & Mudrik, 2018; Faivre & Koch, 2014), but might be considered to be at odds with findings of scene processing in the near absence of attention (e.g., Li et al., 2002). Notably however, in those studies, participants were aware of the stimuli, while in our case, they reported not seeing them in the vast majority of the trials. Thus, the discrepancy in findings is probably related to the difference in the presence/absence of conscious perception, despite the fact that both studies manipulated attention.

Finally, it is surprising that the exploratory analysis of differences between neutral and aversive stimuli yielded no results, both for trials in which participants reported not seeing the stimuli, and in those where they reported seeing them. While the null result for invisible stimuli can simply be taken as evidence against scene processing in the absence of awareness, in line with the lack of difference between the scrambled and the intact stimuli, the null result for the visible stimuli is perplexing given previous findings (Lipp & Derakshan, 2005; Sebastiani et al., 2011; Wiemer et al., 2013). It is possible that the stimuli differed in more than their valence group; in recent years, the IAPS database has been criticized for the stimuli differing in their complexity (e.g., Bradley et al., 2007), having imbalanced representation of humans (overrepresented in the high-arousal group; Colden et al., 2008), and varying in their image quality and low-level features (e.g., Marchewka et al., 2014). As a result, alternative databases for emotion-inducing image sets have been suggested (e.g., Marchewka et al., 2014). Future studies might accordingly benefit from using such databases to explore differences in the processing of emotional stimuli when unconscious.

The paradigm we suggest here is naturally not devoid of limitations; First, there are two potential confounds that could provide an alternative interpretation of our results. One pertains to memory effects, and the other to the resolution of the images we used. We argue that both are mitigated by the data, but future studies should take them into account. The memory concern—inattentional amnesia (Wolfe, 1999)—is common in IB studies. That is, since awareness probes were not presented immediately following the stimulus, participants might have still seen the stimuli, but forgot by the time they were asked about them. Historically, this concern was mitigated by showing that there was no difference in noticing an unexpected event between a condition where the report was provided immediately after the event ended compared with conditions where the report was given tens of seconds later (Becklen & Cervone, 1983). More recently, an IB study with online reporting demonstrated that participants failed to report the unexpected event even while it was still presented, directly demonstrating that the results cannot be explained by a memory failure (Ward & Scholl, 2015). In our work, the amnesia concern is mitigated, at least to some extent, by the lack of relationship between the time that awareness was probed and the time the stimulus appeared (and such a relationship would have been expected if the effect were indeed driven by memory). Nevertheless, it can still be claimed that the lack of relationship between awareness measures and time can stem from a floor effect, as the probes appeared seconds after the last intact instance of the stimulus (and so memory might have already fully decayed). Therefore, the results might still reflect a memory failure rather than a perceptual one. However, the fact that half of the images were aversive (e.g., a bleeding dead person, tumor removal surgery) makes the claim that participants consciously experienced the stimuli but forgot them less likely (some of the scenes were so negative that participants were horrified once presented with the objective probe, surprised to learn that they may have appeared during the trial). Future studies can try to probe participants’ awareness immediately following the last intact presentation of the critical stimulus. The resolution confound, which is unique to this specific setup and is not shared with other IB studies or with future uses of the VRIB paradigm we present here, pertains to the relatively low resolution of the IAPS images used in this study. Potentially, one might claim that the low visibility we found does not stem from the efficacy of the attentional manipulation, but rather from the resolution of the images being too low to be detected with reduced attention. We accordingly conducted a follow-up experiment to directly test this claim, and found evidence that is incompatible with this hypothesis (see Supplementary Data).

A second limitation concerns the fact that while the VRIB method is more ecological than other paradigms, the task itself (tracking a single bee in a dynamic array of bees) does not resemble common day-to-day tasks and processes. Thus, there is still room for improvement with respect to the ecological value of the task, which can potentially benefit from substituting it with a more ecological one. One promising option is to use a driving task, as in the “looked but failed to see” phenomenon (LFBTS; White & Caird, 2010). There, drivers fail to see a target despite looking at it, causing car accidents. Substituting the bee task with a driving task can accordingly provide a new way to study this phenomenon, which has clear and crucial real-life implications.

Finally, designing and conducting VR experiments is more challenging than conventional computer-based experiments; they require costly hardware, and setting them up is more complicated than setting up an experiment on a screen. We do not underestimate this limitation, though we hold this effort to be worthwhile, given the abovementioned advantages that crucially allow us to extend previous research to daily life. Importantly, in the case of the current paradigm, this limitation is substantially mitigated, as the code is shared and can be modified based on the specific needs and research question.

In sum, the VRIB paradigm is a novel technique that can be used to examine the differences between conscious and unconscious processes in an ecologically valid manner. Furthermore, as the method induces repeated IB, the neural correlates of unconscious processes can be inspected with neural imaging techniques that are VR-compatible (such as electroencephalography [EEG]). Thus, the VRIB provides the opportunity to generalize previous conclusions, to provide new means to study their neural underpinnings, and to look further into the relationship between attention and consciousness, in an environment that brings research one step closer to lifelike experiences. In addition, it opens the gate to comparing the neural correlates of real-life conscious and unconscious processing, expanding the existing search for the neural correlates of consciousness (Crick & Koch, 1990) into the ecological domain.

### Supplementary information

Below is the link to the electronic supplementary material.

Supplementary file1 (DOCX 1727 KB)

## Declarations

### Open practices statement

All the collected data from all three experiments, as well as the preregistration for the preregistered experiment, are available on OSF. All analysis materials and the VRIB experiment are available on GitHub.
