# Supplementary material for: Using virtual reality to induce multi-trial inattentional blindness despite trial-by-trial measures of awareness
Source: Behav Res Methods. 2024 Apr 9;56(4):3452–68. doi: 10.3758/s13428-024-02401-8 (PMC11133062; doi:10.3758/s13428-024-02401-8)
Supplement: Supplementary file 1 — Supplementary file1 (DOCX 1727 KB) [file 13428_2024_2401_MOESM1_ESM.docx]

# Supplementary Materials

- OSF pre-registered project link: <https://osf.io/648bp/?view_only=c0993dfb69a9473e95f78a76627735c5>
- Analyses codes and experiment: <https://github.com/RonyHirsch/VRIB_Analysis>
- VRIB paradigm demo video: <https://youtu.be/WrFicpuUqcE>

# Supplementary Data

As we explain in the discussion, it might be claimed that the images presented at bus stops may not have been of high enough resolution to be perceived without focused attention. We accordingly conducted a control experiment to examine this alternative interpretation of our results.

We used an existing paradigm to present the stimuli outside focused attention, and asked if they can nevertheless be detected above chance. To make the test more meaningful, we further degraded the resolution of the stimuli by blurring them. The rationale was that if participants could perform this task even when the resolution is intentionally lower than what was presented in our task, this would be less of a concern as a potential confound explaining our results. We hypothesized that observers would still successfully discriminate the degraded stimuli, despite the low resolution and the attentional manipulation. Such a result would render the proposed explanation less plausible, showing that even at an even lower resolution, and without focused attention, these stimuli can be discriminated.

**Participants**. Participants (N=20; 10 females, aged 18-32, M=26.05, SD=4.19) were recruited via the Prolific platform, based on the following requirements: aged 18-35, a 95% approval rate or higher (as verified by Prolific), and participating via a desktop computer (rather than iPad or phone). They read the instructions and provided their informed consent in Prolific. Then, they were automatically redirected into Pavlovia, an online platform for running Psychopy experiments (Peirce et al., 2019).

**Stimuli**. All stimuli and distractors were identical to the ones used in our manuscript, besides being blurred using Photoshop’s 10-pixel Gaussian blur (Adobe Inc., 2019; Supplementary Figure 1 panel A).

**Procedure**. The task was a modification of one of the tasks in Groen et al.’s work (2016), which examined scene perception in conditions of reduced attention. Participants viewed a stream of black letters, presented at the screen center on a gray background. Each letter was presented for 240ms. Every nine letters on average (uniform distribution between eight and ten), the letter was blue instead of black, and a stimulus image was simultaneously presented behind it for 100ms. Then, participants were asked to report (i) whether the blue letter was the same letter as the previous blue letter (either “yes” or “no”; a 1-back task on the blue letters), and then (ii) select the stimulus image out of an array of four images (a 4AFC task identical to the one in the main study). Importantly, they received immediate trial-by-trial feedback on (i), but not on (ii), as seen in Supplementary Figure 1 (panel B).


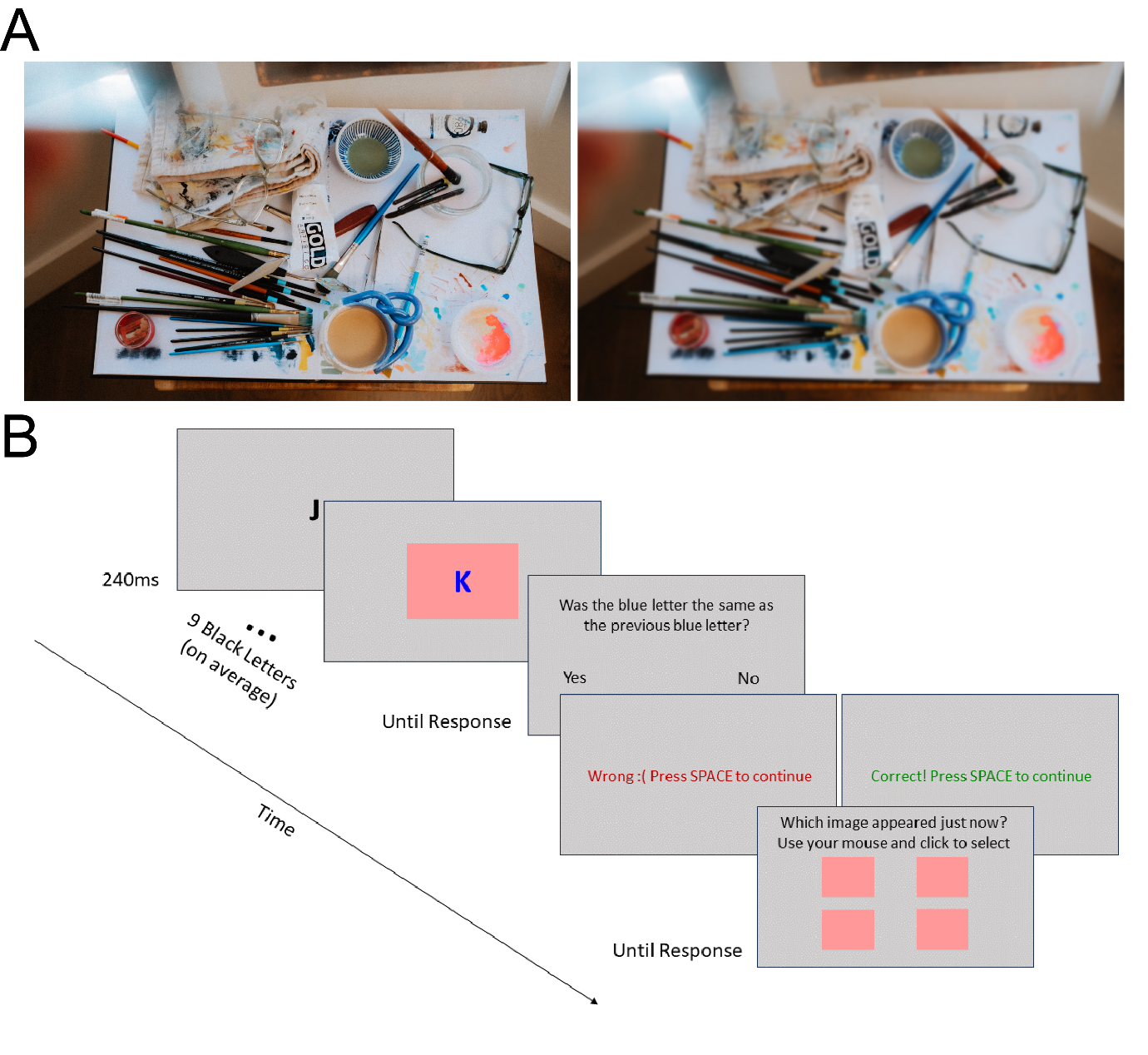


Supplementary Figure 1: Control experiment stimulus manipulation and experimental paradigm. **Panel A**: A 10-pixel Gaussian blur done in Adobe Photoshop, demonstrated here on an image taken from Unsplash for illustration purposes, as IAPS images cannot be publicly shared. Left: the original image. Right: the blurred version. **Panel B**: The experimental paradigm. Black letters appeared one after the other in a stream of nine letters on average. Then, a blue letter appeared, with a simultaneous stimulus image behind it, followed by the 1-back probe. Notably, the location of “Yes” and “No” was counterbalanced within each participant, to avoid automatic responses. Following the response, immediate feedback was given. Then, the 4AFC probe appeared. Once the image was selected, the stream of black letters continued.

**Analysis and Results**. We performed one sample t-tests to examine whether performance in tasks (i) and (ii) was different than chance level (50% and 25%, respectively). We first found that participants were successful in the central task, suggesting that their attention was indeed focused on the letters: they were correct 84.62% of the time on average (SD=14.17), showing better than chance performance (t(19)=10.93, p<0.001, 95% CI=[27.99, 41.26], BF_10_=8.74×10^6^; Supplementary Figure 2). Crucially, they were also successful in discriminating the blurred stimulus image out of an array of four images (M=69.12, SD=18.90, t(19)=10.44, p<0.001, 95% CI=[35.28, 52.97], BF_10_=4.34×10^6^; Supplementary Figure 2). Therefore, despite the dual task and the decreased resolution, participants managed to successfully perceive the stimuli without focused attention. In light of these results, it seems less likely that the chance performance in the very same 4AFC task in the VRIB task solely stems from insufficient resolution of the critical images.


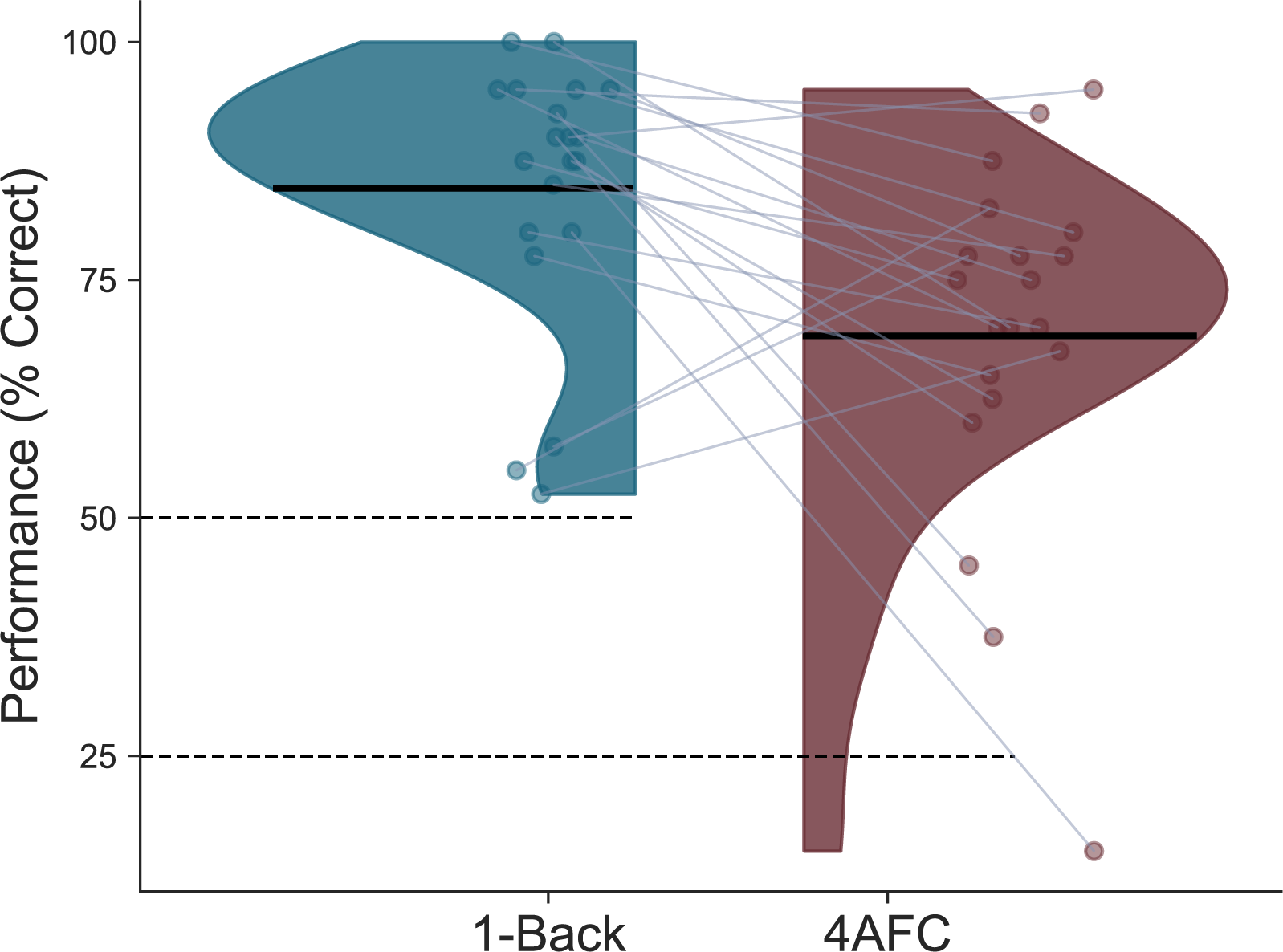

Supplementary Figure 2: Performance in the control experiment tasks. Solid lines denote the average performance, and dashed lines denote chance performance in each task. Individual dots denote participants; gray lines connect pairs of dots representing a single participant. **Left** **(blue)**: Performance in the 1-Back task (determining whether the current blue letter is identical to the previous blue letter). **Right (red)**: Performance in the 4AFC task (selecting the target image).
